# Supplementary material for: Elephant Endotheliotropic Herpesvirus Is Omnipresent in Elephants in European Zoos and an Asian Elephant Range Country
Source: Viruses. 2021 Feb 11;13(2):283. doi: 10.3390/v13020283 (PMC7917619; doi:10.3390/v13020283)
Supplement: Supplementary file 1 [file viruses-13-00283-s001.zip › Hoornweg et al Supplement/Hoornweg et al - Supplementary Figure 1.docx]

**Supplementary Figure 1.**

(A)

HCMV -MRPGLPSYLIILAVCLFSHLLSSRYGAEAVSEPLDKAFHLLLNTYGRPIRFLRENTTQC 59

EEHV1A MRRAAMGRFAAMLQVFVLTDLVSHN-------------------NVMSAFDLSRVHSESC 41

* .: : :* * :::.*:* . . : : * :: .*

HCMV TYNSSLRN-STVVRENAISFNFFQSYNQYYVFHMPRCLFAGPLAEQFLNQVDLTETLERY 118

EEHV1A FKTPELSAETIDLTPNLVIFKFFSNQTHSQVFHLPKCIFDSDLTTYLFKHLDIYEDVTMY 101

. .* : : * : *:**.. .: ***:*:*:* . *: :::::*: * : *

HCMV QQRLNTYALVSKDLASYRSFSQQLKAQDSLGEQPTTVPPPI---DLSI---PHVWMPPQT 172

EEHV1A KNRFEKFYMASVEG-TYKTIIIEGTDNTPYLDQTTAYNPENTVKDLIITYKDMKYMNPYP 160

::*::.: :.* : :*::: : . : :* *: * ** * :* *

HCMV TPHGWTESHTTSGLHRPHFNQTCILFDGHDLL-FSTVTPCLHQGFYLIDELRYVKITLTE 231

EEHV1A I-------------LSLIDDPPCEVFEDIDELILPYFGRCR--RFYLNFDRTVVEGHITS 205

: * :*:. * * : . * *** : *: :*.

HCMV DFFVVTVS-ID--DDTPMLLIFGHLPRVLFKAPYQRDNFILRQTEKHELLVLVKKDQLNR 288

EEHV1A SFVTIYYTSKNGTTPYKIRMFFGNSGDVVYALPFEAQDLSFRMMIREDFQIIGEVAAVKT 265

.*..: : : : ::**: *:: *:: ::: :* :.:: :: : ::

HCMV HSYLKDPDFLDAALDFNYLDLSALLRNSFHRYAV---DVLKSGRC--QMLDRRTVEMAFA 343

EEHV1A MLETFKMDRLDSLLKQNHEDVSNDFKHLFSGFYLHTQQILQGGITRDSLFLEQLLDPLLT 325

. * **: *. *: *:* ::: * : : ::*:.* .:: .: :: ::

HCMV YALALFAAARQEEAGAQVSVPRALDRQAALLQIQEFM------ITCLSQTPPRTTL---- 393

EEHV1A YGIANYVQHRYPYTDKWRGIENVLETETYMYIIPELFELFANNMTIVTPLRPNATKFMDI 385

*.:* :. * :. .: ..*: :: : * *:: :* :: *.:*

HCMV ----LLYPTAVDLAKRALWTPNQITDITSLVRLVYILSKQNQQHLIPQWAL----RQIAD 445

EEHV1A LLNVYSYKSTGPLDHRGLFI--------YFLKFIY--QK-NVTEDVATYAHLYMTKLYRT 434

* :: * :*.*: :::::* .* * . : :* :

HCMV FALKLHKTHLASFLSAFARQELYLMGSLVHSMLVHTTERREIFIVETGLCSLAELSHFTQ 505

EEHV1A YTYPDSKEEETIYKSANDSVDLFILNTIAL-KSGNKTLTRHILLLQTGMCNIKNILGHFH 493

:: * . : : ** :*:::.::. :.* *.*::::**:*.: :: . :

HCMV LLAHPHHEYLSDLYTPCSSSGRRDHSLERLTRLFPDATVPATVPAALSILSTMQPSTLET 565

EEHV1A ILTN-NERKLGNLLSPCFRSLRYDLTETKINELITTKSLQRYG-RLVGMVHHMTK-NSSM 550

:*:: :.. *.:* :** * * * : ::..*: :: :.:: * . .

HCMV FPDLFCLPLGESFSALTVSEHVSYIVTNQYLIKGISYPVSTTVVGQSLIITQTDSQTKCE 625

EEHV1A LNIIKCPLPEDGLSAIVPVEDKLYIVSSKPMATGVVYKGRYTSVSSFIYVTRIQNG-TCV 609

: : * :.:**:. *. ***:.: : .*: * * *.. : :*: :. .*

HCMV LTRNMHTTH----SITVALNISLENCAFCQSALLEYDDTQGVINIMYMHDSDDVLFALDP 681

EEHV1A HIDRIFEEGPLKAVYSLGIDTAKECGDMCPSVLVEYGTNTGFIGLYIITNIEDLTYISKN 669

.:. ::.:: : * :* *.*:**. . *.*.: : : :*: : .

HCMV YNEVVVSSPRTHYLMLLKNGTVLEVTDVVVDATDSRLL-MMSVYALSAIIGIYLLYRMLK 740

EEHV1A ---RKLFPETSHYIWLLKNDTVLELEGTNLFLFSSRSPGAIILYIIIISLIIWTLYEIIK 726

: :**: ****.****: .. : .** : :* : : *: **.::*

HCMV TC----------- 742

EEHV1A LFCYRRQWQYQKL 739

(B)

HCMV ------MCRRPDCGFSFSP-------------GPVILLWCCLLL-PIVSSAAVSVAPTAA 40

EEHV1A MITNVNLMYGPGCNIRKMESTIVTTIIDTLRLGECVTIFTNMLIILLLAESPNKVCASSY 60

: *.*.: * : :: :*: :::.: .*. ::

HCMV EKVPAECPELTRRCLLGEVFEGDKYESWLRPLVNVTGRDGPLSQLIRYRPVTPEAANSVL 100

EEHV1A PHISPSCYNSTLTCLNGGNLSFPG----------MPQYSSNYSKLIRYGYGPNIRTSEYP 110

:: .* : * ** * :. : .. *:**** :..

HCMV LDEAFLDTLALLYNNPDQLRALLTLLSSDTAPRWMTVMRGYSE-------------CGDG 147

EEHV1A IDQKVYDALSLFYRNEEDMRVFLSLR-KDSNGTWEKGLIGVPELKTVQEDERKYVFCDKV 169

:*: . *:*:*:*.* :::*.:*:* .*: * . : * * *..

HCMV SPAVYTCVD--DLC--RGYDLTRLSYGRSIFTEHVLGFELVPPSLFN--VVVAIRNEATR 201

EEHV1A YAT-FYCSPYTKNCNNGKRNLNELPYVDSIFTEHVVEIVFHGSPTLKIEVKILLYNPVTL 228

: : * . * :*..* * *******: : : :: * : : * .*

HCMV TNRAVRLPVSTAAAPEG-IT-LFYGLYNAVKEFCLRHQLDPPLLRHLDKYYA-------- 251

EEHV1A EHRIVTIPLFTPALLDATFNILYRTLYR--------DPTSHALLKTFKNFFDQNIEEPYR 280

:* * :*: * * :. :. *: **. . . **: :.:::

HCMV GLPPELKQTRVNLPAHSRYGPQAVDAR 278

EEHV1A GPKNDRFVRVWQKDGFARVGGPTL--- 304

* : : ..:* * ::

**Supplementary Figure 1 - HCMV strain Merlin and EEHV1A strain Kimba gH and gL protein alignments.** (A) Clustal Omega alignment of HCMV and EEHV1A gH (GenBank accession number YP_081523 and AGG16086, respectively). HCMV gH Cys-95, which is involved in the covalent interaction with gL to form the gH/gL dimer (1), and the corresponding EEHV1A cysteine residue (Cys-78) are highlighted in green. (B) Clustal Omega alignment of HCMV and EEHV1A gL (GenBank accession numbers YP_081555 and AGG16117, respectively). HCMV Cys-47, involved in the covalent interaction with gH (1), and the corresponding cysteine residue in EEHV1A (Cys-67) are highlighted in green.

**References**

1. Ciferri C, Chandramouli S, Donnarumma D, Nikitin PA, Cianfrocco MA, Gerrein R, et al. Structural and biochemical studies of HCMV gH/gL/gO and Pentamer reveal mutually exclusive cell entry complexes. Proceedings of the National Academy of Sciences. 2015;112(6):1767-72.
