# Supplementary material for: Elephant Endotheliotropic Herpesvirus Is Omnipresent in Elephants in European Zoos and an Asian Elephant Range Country
Source: Viruses. 2021 Feb 11;13(2):283. doi: 10.3390/v13020283 (PMC7917619; doi:10.3390/v13020283)
Supplement: Supplementary file 1 [file viruses-13-00283-s001.zip › Hoornweg et al Supplement/Hoornweg et al - Supplementary Figure 1.pdf]

Supplementary Figure 1.

(A)

```
HCMV            -MRPGLPSYLIIILAVCLFSHLLSSRYGAEAVSEPLDKAFHLLLNTYGRPIRFLRENTTQC     59
EEHV1A          MRRAAMGRFAAMLQVFVLTDLVSHN-------------------NVMSAFDLSRVHSESC     41
                  * .:  :  :* * :::.*:* .                   .    : : * :: .*

HCMV            TYNSSLRN-STVVRENAISFNFFQSYNQYYVFHMPRCLFAGPLAEQFLNQVDLTETLERY     118
EEHV1A          FKTPELSAETIDLTPNLVIFKFFSNQTHSQVFHLPKCIFDSDLTTYLFKHLDIYEDVTMY     101
                  . .*   :  :  * : *:**.. .:  ***:*:*:* . *:  :::::*: * :  *

HCMV            QQRLNTYALVSKDLASYRSFSQQLKAQDSLGEQPTTVPPPI---DLSI---PHVWMPPQT     172
EEHV1A          KNRFEKFYMASVEG-TYKTIIIEGTDNTPYLDQTTAYNPENTVKDLIITYKDMKYMNPYP     160
                ::*::.: :.* :  :*:::  : . :    :* *:  *     ** *      :* *

HCMV            TPHGWTESHTTSGLHRPHFNQTCILFDGHDLL-FSTVTPCLHQGFYLIDELRYVKITLTE     231
EEHV1A          I-------------LSLIDDPPCEVFEDIDELILPYFGRCR--RFYLNFDRTVVEGHITS     205
                                   :  * :*:. * * :  .  *    ***  :   *:  :*.

HCMV            DFFVVTVS-ID--DDTPMLLIFGHLPRVLFKAPYQRDNFILRQTEKHELLVLVKKDQLNR     288
EEHV1A          SFVTIYYTSKNGTTPYKIRMFFGNSGDVVYALPFEAQDLSFRMMIREDFQIIGEVAAVKT     265
                .*..:  :  :      : ::**:   *::  *:: ::: :*   :.:: :: :   ::

HCMV            HSYLKDPDFLDAALDFNYLDLSALLRNSFHRYAV---DVLKSGRC--QMLDRRTVEMAFA     343
EEHV1A          MLETFKMDRLDSLLKQNHEDVSNDFKHLFSGFYLHTQQILQGGITRDSLFLEQLLDPLLT     325
                     . * **: *. *: *:*  ::: *  : :   ::*:.*    .:: .: ::  ::

HCMV            YALALFAAARQEEAGAQVSVPRALDRQAALLQIQEFM------ITCLSQTPPRTTL----     393
EEHV1A          YGIANYVQHRYPYTDKWRGIENVLETETYMYIIPELFELFANNMTIVTPLRPNATKFMDI     385
                *.:* :.  *   :.   .: ..*: :: :  * *::      :* ::   *.:*

HCMV            ----LLYPTAVDLAKRALWTPNQITDITSLVRLVYILSKQNQQHLIPQWAL----RQIAD     445
EEHV1A          LLNVYSYKSTGPLDHRGLFI--------YFLKFIY--QK-NVTEDVATYAHLYMTKLYRT     434
                      * ::  * :*.*:          :::::*  .* *  . :  :*     :

HCMV            FALKLHKTHLASFLSAFARQELYLMGSLVHSMLVHTTERREIFIVETGLCSLAELSHFTQ     505
EEHV1A          YTYPDSKEEETIYKSANDSVDLFILNTIAL-KSGNKTLTRHILLLQTGMCNIKNILGHFH     493
                ::    * . : : **    :*:::.::.     :.*  *.*::::**:*.: ::  . :

HCMV            LLAHPHHEYLSDLYTPCSSSGRRDHSLERLTRLFPDATVPATVPAALSILSTMQPSTLET     565
EEHV1A          ILTN-NERKLGNLLSPCFRSLRYDLTETKINELITTKSLQRYG-RLVGMVHHMTK-NSSM     550
                :*:: :.. *.:* :**  * * * :  ::..*:   ::       :.::  *   . .

HCMV            FPDLFCLPLGESFSALTVSEHVSYIVTNQYLIKGISYPVSTTVVGQSLIITQTDSQTKCE     625
EEHV1A          LNIIKCPLPEDGLSAIVPVEDKLYIVSSKPMATGVVYKGRYTSVSSFIYVTRIQNG-TCV     609
                :  : *    :.:**:.  *.  ***:.: : .*: *    * *.. : :*: :.  .*

HCMV            LTRNMHTTH----SITVALNISLENCAFCQSALLEYDDTQGVINIMYMHDSDDVLFALDP     681
EEHV1A          HIDRIFEEGPLKAVYSLGIDTAKECGDMCPSVLVEYGTNTGFIGLYIITNIEDLTYISKN     669
                   .:.         ::.:: : *   :* *.*:**. . *.*.:  : : :*: :  .

HCMV            YNEVVVSSPRTHYLMLLKNGTVLEVTDVVVDATDSRLL-MMSVYALSAIIGIYLLYRMLK     740
EEHV1A          ---RKLFPETSHYIWLLKNDTVLELEGTNLFLFSSRSPGAIILYIIIISLIIWTLYEIIK     726
                     :    :**: ****.****: .. :   .**    : :* :   : *: **.::*

HCMV            TC-----------    742
EEHV1A          LFCYRRQWQYQKL    739
```

(B)

```
HCMV        ------MCRRPDCGFSFSP-------------GPVILLWCCLLL-PIVSSAAVSVAPTAA    40
EEHV1A      MITNVNLMYGPGCNIRKMESTIVTTIIDTLRLGECVTIFTNMLIILLLAESPNKVCASSY    60
                  :   *.*.:                 *  : ::  :*:  :::.:  .*. ::

HCMV        EKVPAECPELTRRCLLGEVFEGDKYESWLRPLVNVTGRDGPLSQLIRYRPVTPEAANSVL    100
EEHV1A      PHISPSCYNSTLTCLNGGNLSFPG----------MPQYSSNYSKLIRYGYGPNIRTSEYP    110
             ::  .* : *  ** *  :.             :   ..  *:****       :..

HCMV        LDEAFLDTLALLYNNPDQLRALLTLLSSDTAPRWMTVMRGYSE-------------CGDG    147
EEHV1A      IDQKVYDALSLFYRNEEDMRVFLSLR-KDSNGTWEKGLIGVPELKTVQEDERKYVFCDKV    169
            :*: . *:*:*:*.* :::*.:*:*  .*:   * . : *  *             *..

HCMV        SPAVYTCVD--DLC--RGYDLTRLSYGRSIFTEHVLGFELVPPSLFN--VVVAIRNEATR    201
EEHV1A      YAT-FYCSPYTKNCNNGKRNLNELPYVDSIFTEHVVEIVFHGSPTLKIEVKILLYNPVTL    228
              : : *    . *     :*..* *  *******: : :     ::  * : : * .*

HCMV        TNRAVRLPVSTAAAPEG-IT-LFYGLYNAVKEFCLRHQLDPPLLRHLDKYYA--------    251
EEHV1A      EHRIVTIPLFTPALLDATFNILYRTLYR--------DPTSHALLKTFKNFFDQNIEEPYR    280
             :* * :*: * *  :. :. *:  **.        .  .  **: :.:::

HCMV        GLPPELKQTRVNLPAHSRYGPQAVDAR         278
EEHV1A      GPKNDRFVRVWQKDGFARVGGPTL---         304
            *   :      :  ..:* *  ::
```

Supplementary Figure 1 - HCMV strain Merlin and EEHV1A strain Kimba gH and gL protein alignments. (A) Clustal Omega alignment of HCMV and EEHV1A gH (GenBank accession number YP_081523 and AGG16086, respectively). HCMV gH Cys-95, which is involved in the covalent interaction with gL to form the gH/gL dimer (1), and the corresponding EEHV1A cysteine residue (Cys-78) are highlighted in green. (B) Clustal Omega alignment of HCMV and EEHV1A gL (GenBank accession numbers YP_081555 and AGG16117, respectively). HCMV Cys-47, involved in the covalent interaction with gH (1), and the corresponding cysteine residue in EEHV1A (Cys-67) are highlighted in green.

References

1. Ciferri C, Chandramouli S, Donnarumma D, Nikitin PA, Cianfrocco MA, Gerrein R, et al. Structural and biochemical studies of HCMV gH/gL/gO and Pentamer reveal mutually exclusive cell entry complexes. Proceedings of the National Academy of Sciences. 2015;112(6):1767-72.
